# Calibration of discrete element parameters for simulating wheat crushing

**DOI:** 10.1002/fsn3.3693

**Published:** 2023-09-20

**Authors:** Xuefeng Wang, Wenbin Wu, Huapo Jia

**Affiliations:** ^1^ School of Mechanical and Electrical Engineering Henan University of Technology Zhengzhou China; ^2^ School of Mechanical Engineering Zhengzhou University of Science and Technology Zhengzhou China

**Keywords:** compression, DEM, parameter calibration, wheat

## Abstract

The mechanical properties of wheat grains were measured and analyzed using discrete element software, which provided crucial data for their processing in a mill. The foundational Hertz–Mindlin model was used as a theoretical framework to evaluate the accumulation angle of wheat grains. The Plackett–Burman, steepest ascent, and Box–Behnken methods were utilized in a series of experiments, with the accumulation angle serving as the dependent variable. Targeting the actual angle of repose in the response surface, the optimal parameters were derived using the regression equations. These included a static‐friction coefficient of 0.3 between individual wheat grains, rolling‐friction coefficient of 0.04 for wheat–wheat interactions, static‐friction coefficient of 0.554 for wheat–tooth roller interactions, collision recovery coefficient of 0.5 for wheat–wheat collisions, collision recovery coefficient of 0.45 for wheat–tooth roller collisions, and rolling‐friction coefficient of 0.05 for wheat–roller interactions. Relying on the bonding contact model of Hertz–Mindlin, virtual uniaxial compression tests were conducted to calibrate the wheat grain bonding parameters. A regression equation for the critical load was subsequently generated using the critical load of the wheat grain bonding model as the response variable. The optimal parameters were calculated and incorporated into the EDEM model for computation, which resulted in a relative error of 1.6% between the calculated and observed values, confirming the accuracy and feasibility of the calibration method, suggesting that the calibrated parameters were accurate.

## Graphical abstract

The mechanical properties of wheat grains were measured and analyzed using discrete element software, which provided crucial data for their processing in a mill. The optimal parameters were calculated and incorporated into the EDEM model for computation, which resulted in a relative error of 1.6% between the calculated and observed values, confirming the accuracy and feasibility of the calibration method, suggesting that the calibrated parameters were accurate.

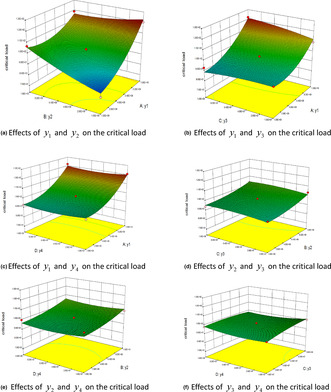

**Practical application**

This study primarily focused on the calibration of parameters for simulating wheat grain processing in discrete element software. The discrete element method is a crucial approach in simulating wheat processing, wherein the precision of the wheat's mechanical parameters significantly influences the outcome of the simulations. This study mainly investigated the mechanical property parameters of wheat, laying the foundation for studying wheat milling in flour production. Thus, the research reported in this paper holds substantial importance.

## 1 INTRODUCTION

This study primarily concentrated on calibrating the parameters for wheat grain processing simulation in discrete element software. A mill is the central piece of machinery in the wheat‐milling process, which is influenced by the combined effects of extrusion, shear, friction, and rubbing. The non‐visual nature of wheat processing poses significant challenges in understanding the milling mechanism within the mill. In 2016, Patwa et al. (2016) suggested that the discrete element method might provide a superior way to study the milling mechanism within a mill. The EDEM software allows for the visualization of the entire wheat‐crushing process. The discrete element method (DEM) introduced by Cundall in 1979 (Cundall & Strack, 1979; Reeves, 2013; Zhong et al., 2016) has been widely applied in diverse fields, including the agriculture, food, chemical, and pharmaceutical industries. When using simulation to study the wheat‐milling process, the physical and contact parameters of wheat are critical to understanding its mechanical properties. Consequently, researching these parameters prior to analyzing the milling mechanism is of utmost importance. Currently, these parameters are acquired primarily through direct measurement and virtual calibration. Wang et al. (2012) measured the recovery coefficients of wheat and rapeseed at various moisture levels based on bounce tests. Liu et al. (2016) utilized a Vernier caliper to measure the long and short axes of wheat grains, treating them as regular ellipsoids. The parameters of the wheat's discrete element model were calibrated using five‐ball combinations. However, the wheat's bonding parameters were not examined. Zhang et al. (2020) employed three‐dimensional scanning and reverse engineering methods to capture the contours of grains of rice and created balls with different radii for discrete element models to calibrate the static‐friction and dynamic‐friction coefficients between grains. However, the accuracy of three‐dimensional laser scanning for smaller objects is limited, making it incapable of fully capturing the three‐dimensional contour of the object. Ying et al. (2018) developed a discrete element model of a wheat grain based on X‐ray technology, and calibrated the discrete element parameters of wheat. However, several studies (Chung & Ooi, 2006; Favier et al., 1999; Zeebroeck et al., 2003) have indicated that increasing the particle accuracy significantly extends the simulation time. Furthermore, numerous studies (Härtl, 2008; Markauskas et al., 2010; Pasha et al., 2016) have suggested that an accurate particle shape model does not significantly enhance the simulation accuracy. A rough, non‐spherical model constructed with a limited number of spherical units usually offers better model accuracy.

In this study, building upon global research and using wheat particles as the subject, a discrete element model of wheat grains was developed. A cylindrical lifting experiment was conducted to determine the angle of repose of wheat particles. The Plackett–Burman, steepest ascent, and Box–Behnken experimental designs were employed to calibrate the parameters of the discrete element simulation. A comparison between the simulation values and actual experimental results was made, leading to the acquisition of parameters for the discrete element calibration of wheat particles through simulation. Incorporating Hertz–Mindlin's bond theory, and in conjunction with a compressive failure test on wheat particles, a response surface analysis was used to determine bond parameters, such as the normal contact stiffness, tangential contact stiffness, critical normal stress, and critical tangential stress. This will contribute to future research on the mechanism of wheat milling.

## 2 EXPERIMENTS AND METHODS OF CONTACT PARAMETER CALIBRATION

### 2.1 Experiment model

**FIGURE 1:**
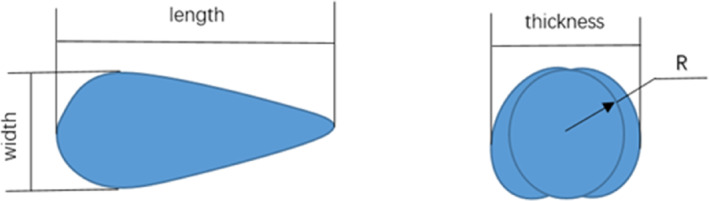
Size and sketch of a wheat particle.

This study considered three varieties of wheat: Zhengmai379, Xinmai26, and Zhoumai36. These varieties are among the most common and representative in China. The moisture content of the wheat grains was determined to be 15.87% (wet basis reference) using a halogen moisture analyzer, which met the necessary condition for wheat processing. A total of 100 wheat particles were randomly and independently selected (Xu et al., 2023), and their dimensions (length, width, and thickness) were measured using a caliper (with a resolution of 0.01 mm). The average dimensions were determined to be 6.15, 3.06, and 2.9 mm for the length, width, and thickness, respectively. The measured results are presented in Figure 1 and Table 1.

**TABLE 1:** Statistical analysis results of wheat triaxial dimensions.

Parameters	Maximum	Minimum	Average	SD
Length (mm)	7.60	4.03	6.15	0.35
Width (mm)	4.05	2.26	3.06	0.31
Thickness (mm)	3.73	2.39	2.90	0.23

To simplify the calculations and reduce the computation time, the equivalent diameter of non‐spherical particles was employed, despite the wheat grains being triaxially non‐uniform particles. The volumetric diameter was calculated using the formula outlined by Xu et al. (2023).

(1) $$d_{v}={\left(\frac{6v}{\pi }\right)}^{\;1/3}$$

**FIGURE 2:**
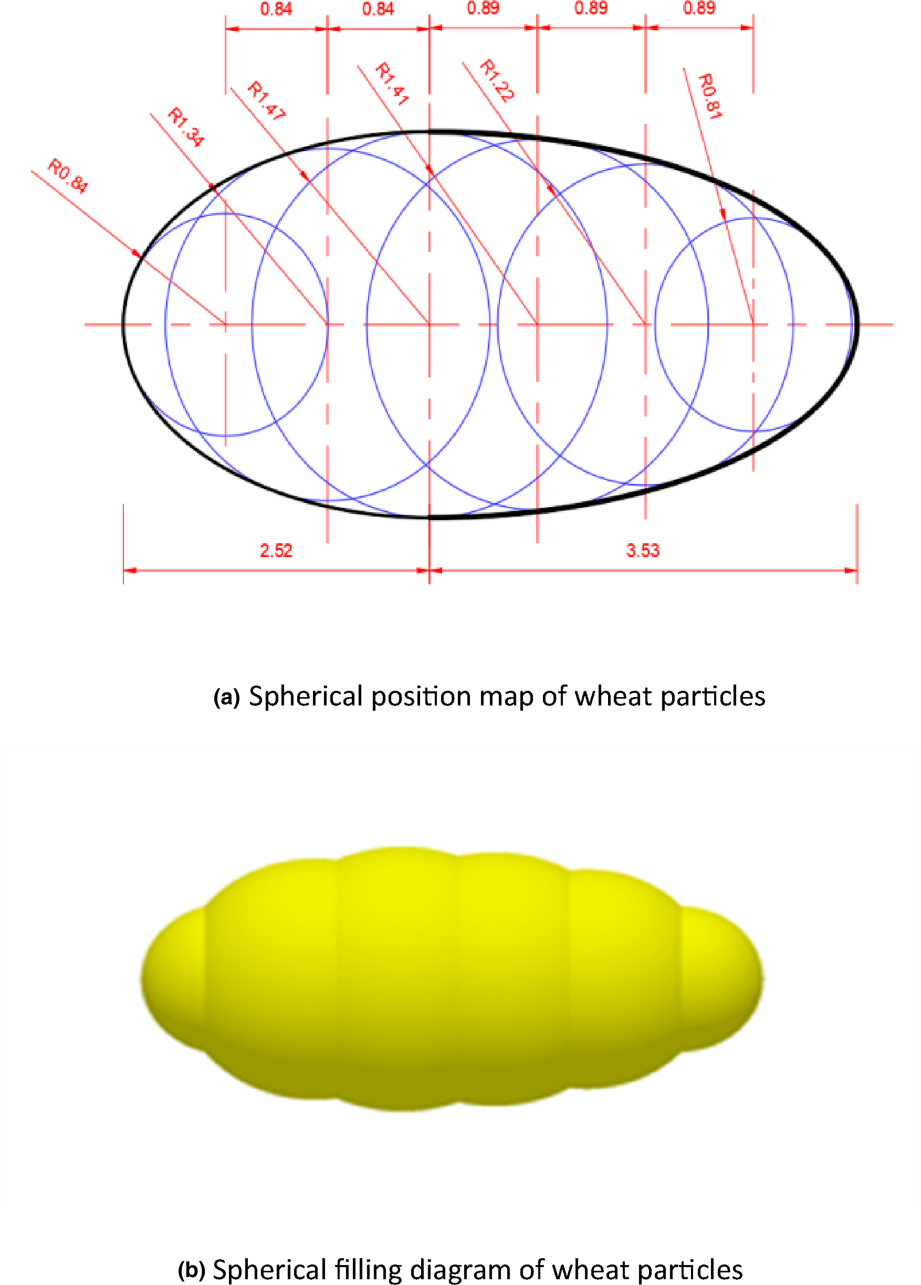
Model of wheat seed for discrete element. (a) Spherical position map of wheat particles. (b) Spherical filling diagram of wheat particles.

As observed from the three‐axis size measurements of the wheat kernels listed in Table 1, the mean value for the length of the wheat kernels was 6.15 mm, which was used for the length of the ellipsoid. The mean values for the widths and thicknesses of the wheat kernels were replaced by a circle with a diameter of 3.00 mm (radius *R* = 1.50 mm). Wheat particles were modeled in the Solidworks software, and the established wheat model was imported into the EDEM software. Based on this, the true shape of wheat grains was approximated, and a simulation model of the wheat grain was constructed using a five‐ball combination, as shown in Figure 2.

**FIGURE 3:**
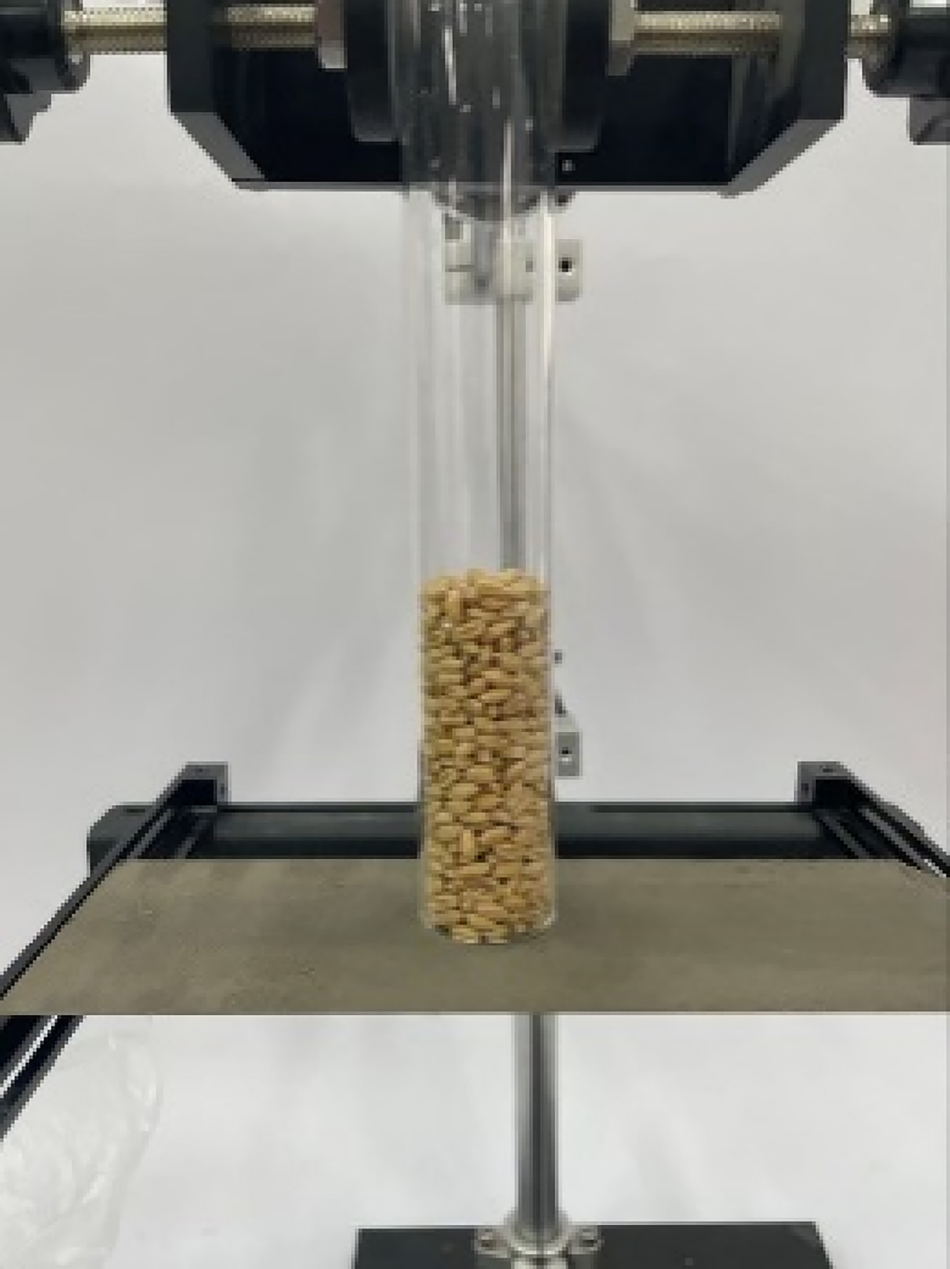
Experimental device for cylinder lifting.

In this study, the cylinder lifting method was employed to measure the angle of repose of wheat. Considering the size of the wheat particles, a cylinder with an inner diameter of 27 mm and a height of 190 mm was used. For the measurement, the cylinder was placed on the horizontal fixed plate of a grinding roller, filled with 30 g of wheat particles, and then slowly elevated at a speed of 0.01 m/s, allowing the particles to accumulate on the grinding roller's flat surface. This experiment was repeated 10 times to obtain the angle of repose of the wheat grains. The experimental setup is illustrated in Figure 3.

**FIGURE 4:**
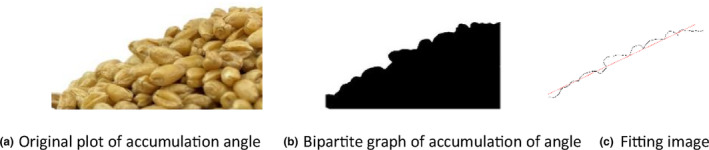
Fitting image of accumulated angle. (a) Original plot of accumulation angle. (b) Bipartite graph of accumulation of angle. (c) Fitting image.

A computer image processing technology was used to quantify the angle of repose more accurately. The accumulated particle image was processed in Matlab software, with the image's boundary pixels acquired via grayscale conversion, threshold segmentation, and boundary searching. Finally, the accumulated angle of the particles was obtained by linear fitting. The angle of repose was 31.74°, as shown in Figure 4.

### 2.2 Simulation experiment

#### 2.2.1 Simulation parameters

The approximate ranges of the simulation parameters in this study were preliminarily determined based on the relevant parameters of the wheat particles and materials of the grinding roller in the discrete element simulation (Akkoyun & Arslan, 2020; Boac et al., 2009; Frączek et al., 2007; Jia et al., 2014), and are listed in Table 2.

**TABLE 2:** Parameters required in DEM simulation.

Simulation parameters	Value
Poisson's ratio of wheat	0.16–0.42
Poisson's ratio of grinder roll	0.3
Shear modulus of wheat/MPa	4.2–997.9
Shear modulus of grinder roll/MPa	2000
Density of acrylic/(kg m^−3^)	1350
Density of grinder roll/(kg m^−3^)	7860
Wheat–wheat restitution coefficient	0.1–0.9
Wheat–acrylic static friction coefficient	0.1–0.6
Wheat–wheat rolling friction coefficient	0–0.1
Wheat–grinder roll restitution coefficient	0.1–0.8
Wheat–grinder roll static friction coefficient	0.3–0.8
Wheat–grinder roll rolling friction coefficient	0–0.1

#### 2.2.2 Simulation model of cylinder lifting

**FIGURE 5:**
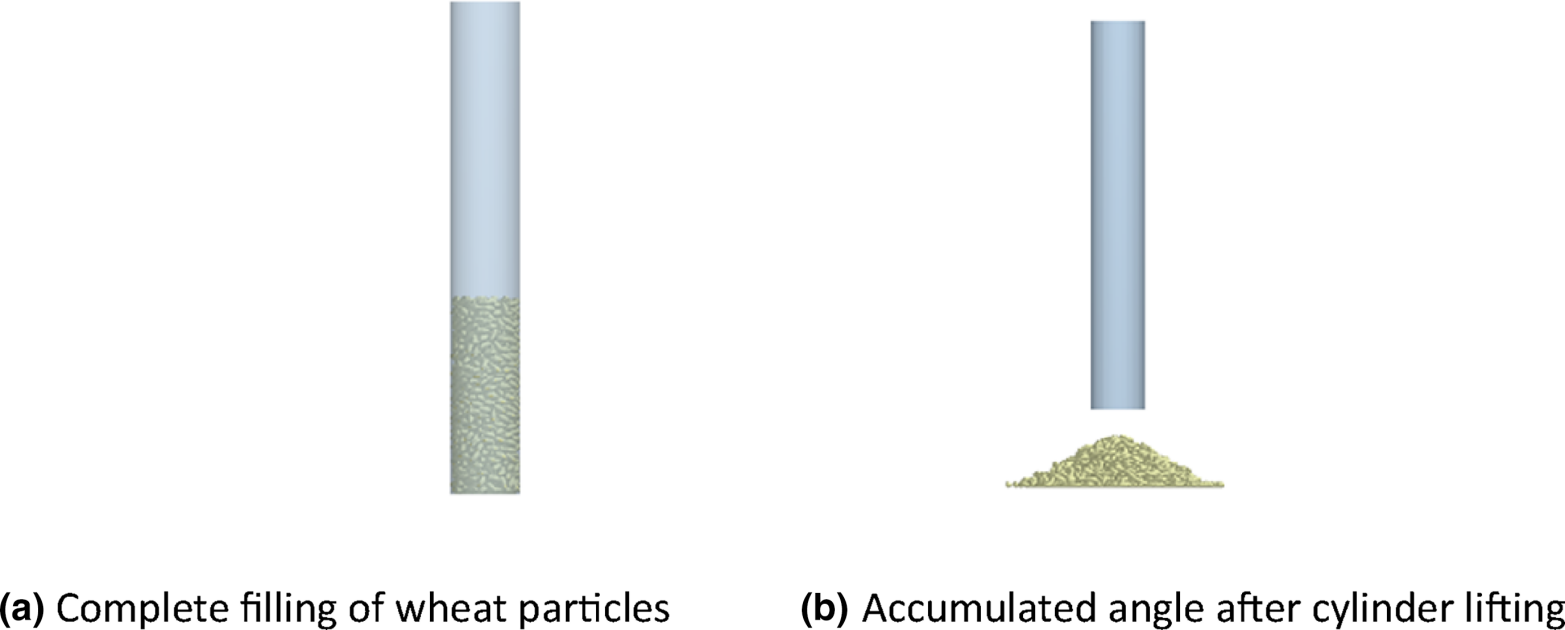
Simulation process diagram. (a) Complete filling of wheat particles. (b) Accumulated angle after cylinder lifting.

The simulation model agreed with the experimental model. A particle factory was established at the top of the cylinder to generate a particle mass of 30 g, allowing the particles to fall freely. After all the particles reached the specified position and remained stationary, the cylinder was raised upward at a speed of 0.01 m/s until all the wheat particles fell off the cylinder and formed a stable pile, as shown in Figure 5.

In this study, the built‐in Hertz–Mindlin (no‐slip) contact model in EDEM was utilized for computations. Given the significant volume of calculations, all the simulation parameters were chosen to have a Rayleigh timestep of 20%. Moreover, the size of the grid in the simulation was set to be three times the size of the smallest spherical element.

### 2.3 Response surface design of the simulation model

#### 2.3.1 Plackett–Burman test

Leveraging the angle of inclination formed by wheat seeds as the response value, the Plackett–Burman test was employed to filter out factors with significant influence on the test and eliminate factors with little impact on the experimental results. This approach reduced the number of experiments and ensured the accuracy of the experimental test results. Six factors affecting the test indices in the cylinder lifting test were selected for examination, and the Plackett–Burman test factor table is presented in Table 3. According to the Plackett–Burman test principle, 12 groups of tests were designed, and the angle of repose for each test group was documented. The design and results of the simulated test are illustrated in Table 4.

**TABLE 3:** The Plackett–Burman test of the factor table.

Factor	Level	
	Low level (−1)	High level (1)
Wheat–wheat collision recovery coefficient *x*_1_	0.1	0.9
Wheat–wheat static friction coefficient *x*_2_	0.1	0.6
Rolling friction coefficient of wheat–wheat *x*_3_	0	0.1
Collision recovery coefficient of wheat–roller *x*_4_	0.1	0.8
Static friction coefficient of wheat– roller *x*_5_	0.3	0.8
Rolling friction coefficient of wheat–roller *x*_6_	0	0.1

**TABLE 4:** The Plackett–Burman trial design and results.

Number	*x*_1_	*x*_2_	*x*_3_	*x*_4_	*x*_5_	*x*_6_	Angle of repose (°)
1	0.9	0.1	0.1	0.1	0.3	0	23.52
2	0.9	0.6	0	0.8	0.3	0	25.05
3	0.1	0.6	0.1	0.1	0.8	0	44.83
4	0.9	0.1	0.1	0.8	0.3	0.1	26.94
5	0.9	0.6	0	0.8	0.8	0	33.04
6	0.9	0.6	0.1	0.1	0.8	0.1	40.08
7	0.1	0.6	0.1	0.8	0.3	0.1	39.76
8	0.1	0.1	0.1	0.8	0.8	0	29.75
9	0.1	0.1	0	0.8	0.8	0.1	18.73
10	0.9	0.1	0	0.1	0.8	0.1	15.99
11	0.1	0.6	0	0.1	0.3	0.1	31.81
12	0.1	0.1	0	0.1	0.3	0	8.57

**FIGURE 6:**
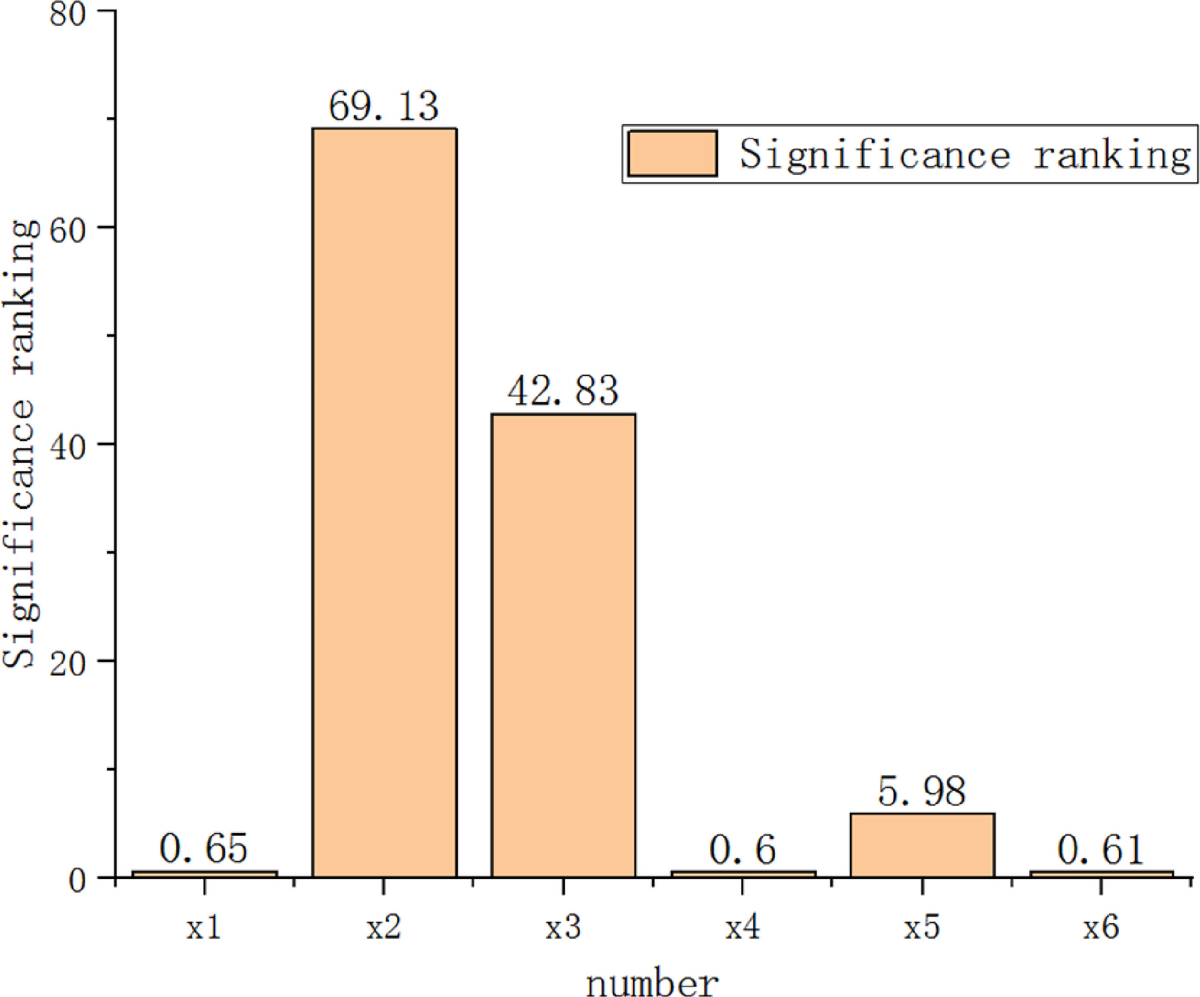
Significance of Plackett–Burman test parameter.

The results of the Plackett–Burman test were analyzed to determine the dominance of each factor, as shown in Figure 6. This analysis provided insight into the impact of each factor on the angle of repose of the wheat particles. Among the factors, the static‐friction coefficient for wheat–wheat collisions, rolling‐friction coefficient for wheat–wheat interaction, and static‐friction coefficient for wheat–grinding roller interaction were dominant, with a confidence interval of 95%. The influence of other factors on the angle of accumulation was insignificant.

#### 2.3.2 Steepest climbing test

To determine the optimal ranges for the dominant factors from the Plackett–Burman test and provide a foundation for subsequent testing, the steepest ascent test was necessary. The Plackett–Burman test identified three dominant factors. Six gradient points were considered for these dominant factors, while median values were used for the non‐dominant factors. A total of six tests were designed, and the angle of repose was recorded for each. Error values were calculated according to Formula (2). The design and results of the steepest ascent test are presented in Table 5.

(2) $$y_{1}=\left|\frac{\alpha -\alpha_{1}}{\alpha_{1}}\right|\times 100\%$$

**TABLE 5:** The results of design of climbing experiment.

Number	*x*_1_	*x*_2_	*x*_3_	*x*_4_	*x*_5_	*x*_6_	Angle of repose (°)	Relative error (%)
1	0.5	0.1	0	0.45	0.3	0.05	14.005	0.56
2	0.5	0.2	0.02	0.45	0.4	0.05	27.19	0.14
3	0.5	0.3	0.04	0.45	0.5	0.05	34.26	0.08
4	0.5	0.4	0.06	0.45	0.6	0.05	38.005	0.20
5	0.5	0.5	0.08	0.45	0.7	0.05	40.53	0.28
6	0.5	0.6	0.1	0.45	0.8	0.05	41.485	0.31

*y*_1_—Relative error, unit (%).

*α*_1_—Angle of the steepest climbing test, unit (°).

*α*—Measured angle of repose, unit (°).

As listed in Table 5, as the static‐friction coefficient of the wheat–wheat interaction, rolling‐friction coefficient of the wheat–wheat interaction, and static‐friction coefficient of the wheat‐grinding roller interaction increased, the relative error initially decreased and then increased. The smallest relative error was observed in the third group test. Therefore, the optimal value ranges for the test factors could be selected around the third group test, that is, between the second group and fourth group tests. The parameters from the third group test were selected as the center points, with the parameters from the second and fourth group tests selected as high and low levels, respectively, for the Box–Behnken test.

#### 2.3.3 Box–Behnken test and regression model

The Box–Behnken test was designed using the results of the Plackett–Burman test and steepest ascent test, and 15 experiments were conducted using the Box–Behnken design principle. The angle of repose of each test was recorded. The Box–Behnken design and results are listed in Table 6.

**TABLE 6:** Box–Behnken trial design and results.

Number	*x*_2_	*x*_3_	*x*_5_	Angle of repose (°)
1	0.2	0.02	0.5	27.06
2	0.4	0.02	0.5	35.84
3	0.2	0.06	0.5	33.25
4	0.4	0.06	0.5	37.39
5	0.2	0.04	0.4	30.17
6	0.4	0.04	0.4	34.88
7	0.2	0.04	0.6	27.65
8	0.4	0.04	0.6	33.91
9	0.3	0.02	0.4	30.39
10	0.3	0.06	0.4	35.28
11	0.3	0.02	0.6	28.11
12	0.3	0.06	0.6	32.56
13	0.3	0.04	0.5	33.34
14	0.3	0.04	0.5	32.84
15	0.3	0.04	0.5	32.05

After analyzing the experimental results, a quadratic regression model was established to illustrate the influences of the parameters for the contact between the wheat and grinding roller on the angle of repose. Three substantial factors were also identified. This regression model is depicted in Formula (3).
(3)

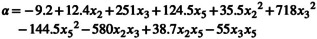




The results of a variance analysis of the regression equation are presented in Table 7. Specifically, a value of *p* < .05 for the fitted model signified that the regression equation was a good fit. The static‐friction coefficient of the wheat–wheat interaction and rolling‐friction coefficient of wheat–wheat interaction had values of less than 0.01, indicating that these two factors significantly affected the angle of repose of the wheat particles. Meanwhile, the quadratic terms of the static‐friction coefficient of the wheat–grinding roller interaction and the static‐friction coefficient of the wheat–grinding roller interaction had values of less than 0.05 and greater than 0.01, respectively. This indicated that these two factors significantly impacted the angle of repose of the wheat particles.

**TABLE 7:** Regression equation analysis of variance.

Sources	DOF	Squares	Mean square	*F*‐Value	*p*‐Value
Model	9	131.866	14.6518	38.03	<0.0001***
*x*_2_	1	71.342	71.3415	185.17	<0.0001***
*x*_3_	1	36.466	36.4658	94.65	<0.0001***
*x*_5_	1	9.01	9.01	23.39	0.005**
${x_{2}}^{2}$	1	0.464	0.4642	1.2	0.322
${x_{3}}^{2}$	1	0.304	0.3043	0.79	0.415
${x_{5}}^{2}$	1	7.714	7.7141	20.02	0.007**
*x*_2_ *x*_3_	1	5.382	5.3824	13.97	0.013*
*x*_2_ *x*_5_	1	0.601	0.6006	1.56	0.267
*x*_3_ *x*_5_	1	0.048	0.0484	0.13	0.737
Error	5	1.926	0.3853		
Lack of fit	3	1.08	0.3601	0.85	0.58
Pure error	2	0.846	0.423		
Sum	14	133.792			

#### 2.3.4 Response surface analysis

**FIGURE 7:**
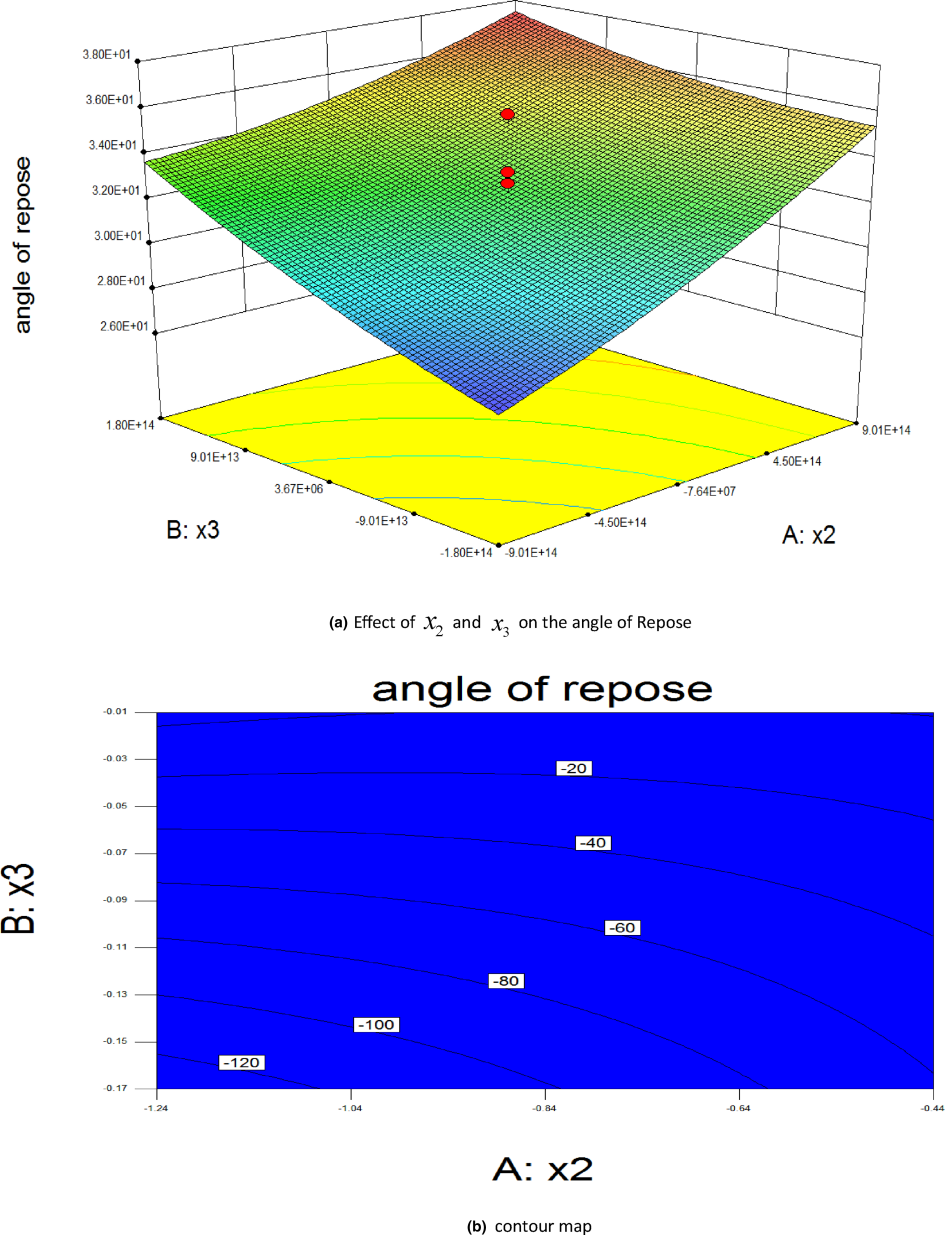
Effects of various factors on the angle of repose. (a) Effect of *x*_2_ and *x*_3_ on the angle of Repose. (b) Contour map.

The response surface was generated to analyze the effects of the static‐friction coefficient of the wheat–wheat interaction, rolling‐friction coefficient of the wheat–wheat interaction, and static‐friction coefficient of the wheat–grinding roller interaction on the angle of repose, and the results are shown in Figure 7.

The regression equation was used to calculate the optimal parameters with respect to the actual angle of repose: the optimal static‐friction coefficient for the wheat–wheat interaction was 0.3, the optimal rolling‐friction coefficient for the wheat–wheat interaction was 0.04, and the optimal static‐friction coefficient for the wheat–grinding roller interaction was 0.554. These optimal parameters were then incorporated into the EDEM software to verify the simulation test. After conducting five verification experiments, the error rate between the simulated angle of repose and actual experimental angle of repose was found to be only 0.9%. This result corroborated the reliability of the parameter calibration, thus confirming its suitability for studying the wheat‐milling process and defining the simulation parameters.

## 3 EXPERIMENTAL CALIBRATION AND METHOD FOR BONDING PARAMETERS

### 3.1 Establishment of bonding model

The bonding model was introduced via the API function of the discrete element software. The bonding model could bond the small particles and prevent normal and tangential movement between them (Liu et al., 2018; Obermayr et al., 2013; Potyondy, 2007). When the force between the small particles exceeded the maximum normal contact stress or maximum tangential contact stress of the bonding contact model, the bond broke. This separation of small particles signaled the fracture of the wheat particles. The calculation for this scenario is expressed in Formula (4).

(4) $$\left\{\begin{array}{c} \delta F_{n}=-v_{n}S_{n}A\delta_{t};\\ \delta F_{t}=-v_{t}S_{t}A\delta_{t};\\ \delta T_{n}=-w_{n}S_{n}J\delta_{t};\\ \delta T_{t}=-w_{n}S_{n}J\delta_{t} \end{array}\right.$$

The maximum normal and tangential stress values are defined in Formula (5),

(5) $$\left\{\begin{array}{c} \sigma_{\max}<\frac{-F_{n}}{A}+\frac{2T_{t}}{J}R_{B};\\ \tau_{\max}<\frac{-F_{t}}{A}+\frac{2T_{n}}{J}R_{B}; \end{array}\right.$$

where *A* denotes the contact area, $A=\pi {R_{B}}^{2}$; $R_{B}$ denotes the bonding radius; *J* denotes the moment of inertia of the particle, $J=0.5\pi {R_{B}}^{4}$; *S*_*n*_ and *S*_*t*_ denote the normal and tangential stiffness values of particles, respectively; *δ*_*t*_ denotes the step size; *v*_*n*_ and *v*_*t*_ denote the normal and tangential velocities of particles, respectively; and *w*_*n*_ and *w*_*t*_ denote the normal and tangential angular velocities of particles, respectively.

**FIGURE 8:**
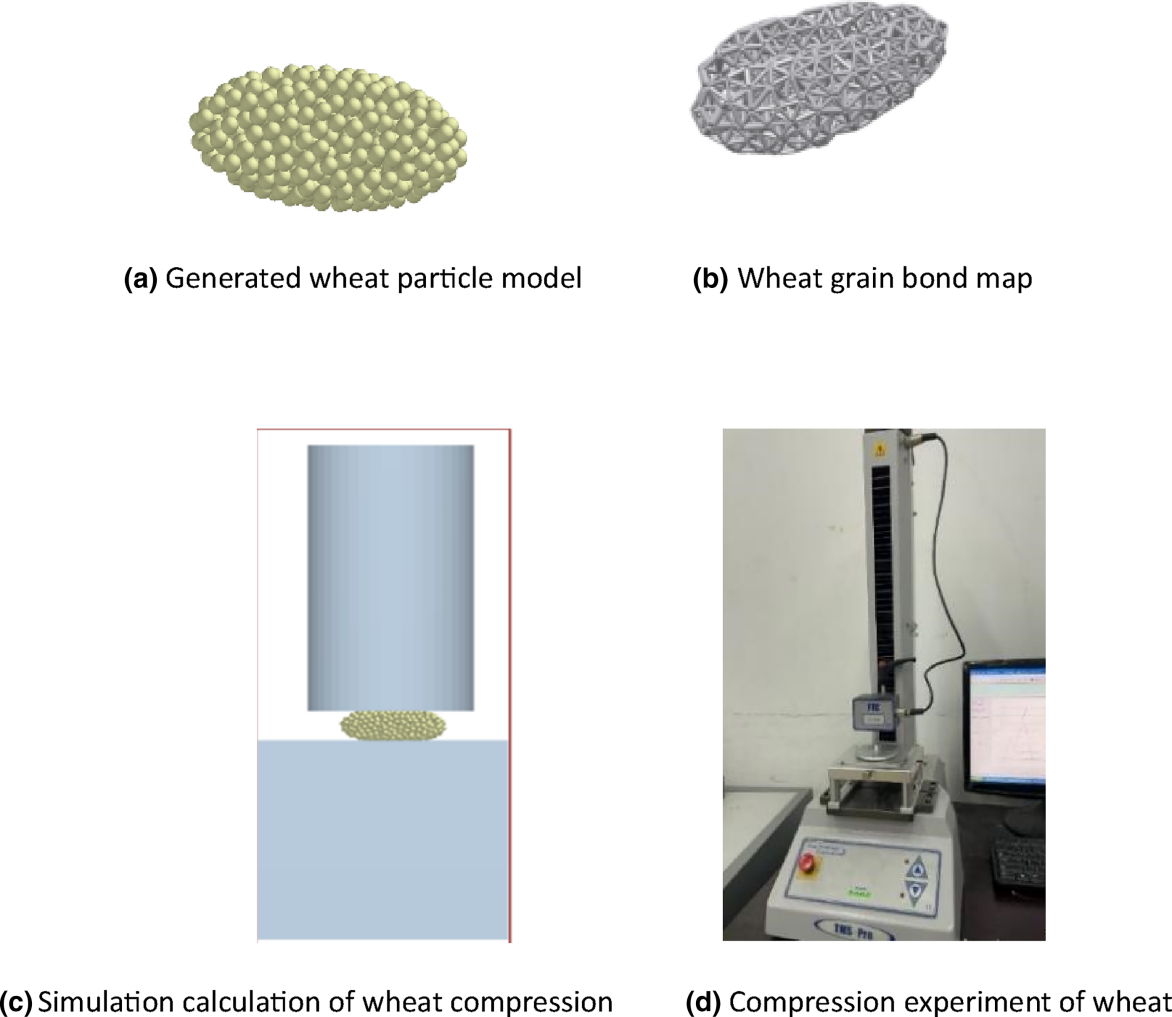
Experiment and simulation of wheat extrusion. (a) Generated wheat particle model. (b) Wheat grain bond map. (c) Simulation calculation of wheat compression. (d) Compression experiment of wheat.

**FIGURE 9:**
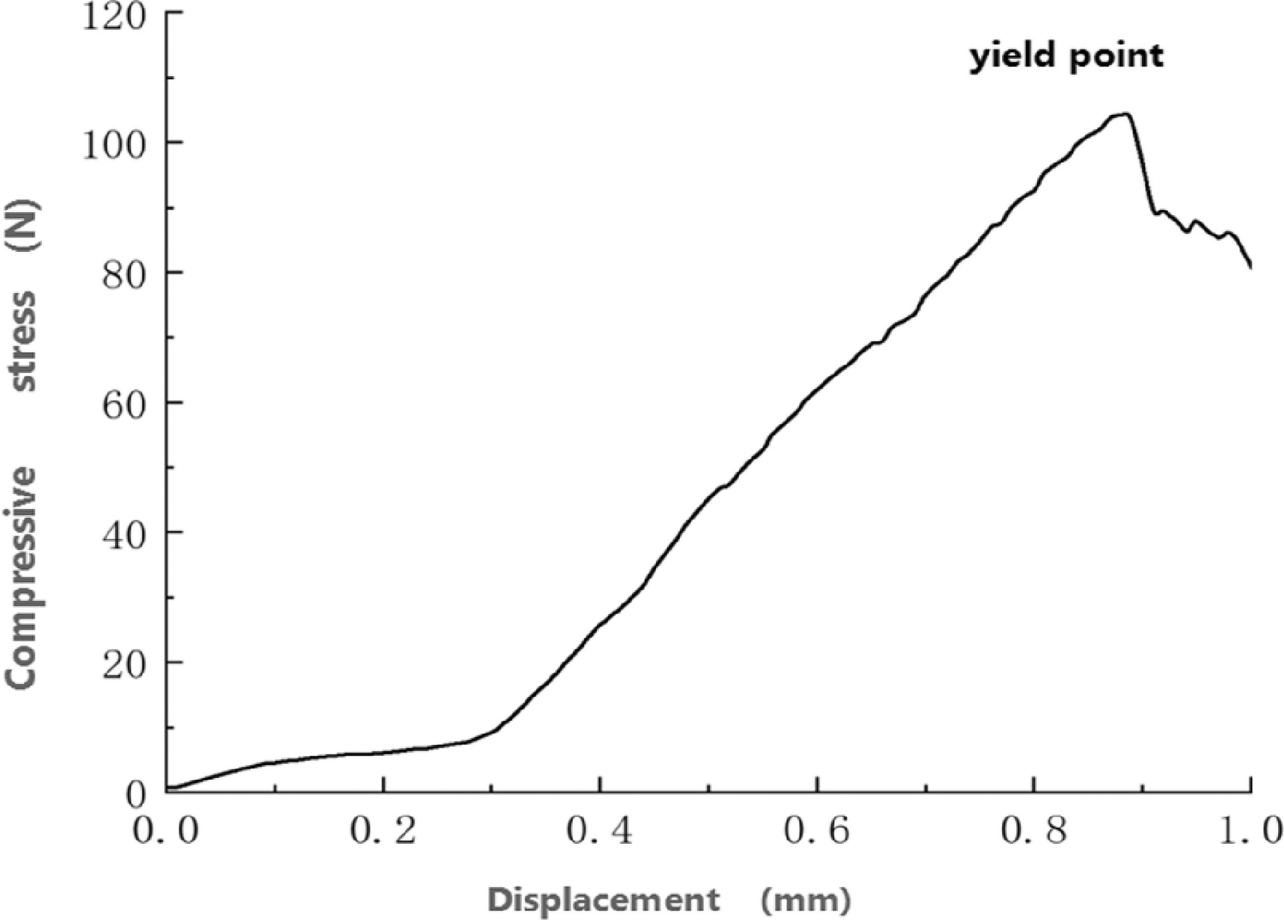
Force–displacement curve of compression of wheat grain.

According to the principles underlying the bond model, the rupture of the bonds between particles was related to parameters, such as the normal contact stiffness, tangential contact stiffness, critical normal stress, critical tangential stress, and bond radius of the wheat particles. To simulate the compressive failure of the wheat more accurately, we generated 512 particles by adding small particles with a diameter of 0.2 mm to the original wheat model. The upper compression plate was then pressed downward at a rate of 6.6 mm/min. The generated wheat particles and compressed wheat are shown in Figure 8. Specifically, Figure 8d represents the TMS‐PRO system, which can test the mechanical characteristics of grain, obtaining the destructive force, destructive energy, force–displacement curves, elastic modulus, and more. The force–displacement curve of the wheat grain compression process is shown in Figure 9. As shown in Figure 9, the wheat grains exhibited yield points during the compression process. Prior to reaching the yield point, the pressure incrementally increased with the compression displacement, and dropped sharply after the yield point was reached.

### 3.2 Results and analysis of bonding model

To determine the optimal bonding parameters of wheat particles, we designed a Box–Behnken experiment to obtain a regression equation that captured the influences of these factors on the relative error rate. Building upon related research (Huang et al., 2014; Oladunmoye et al., 2014; Zeng et al., 2018), we initially selected the bonding parameters listed in Table 8 for the wheat.

**TABLE 8:** Test factors.

Factor	Level	
	Low level (−1)	High level (1)
Normal contact stiffness *y*_1_/(N/m^3^)	10^9^	10^10^
Tangential contact stiffness *y*_2_/(N/m^3^)	10^9^	10^10^
Critical normal stress *y*_3_/(Pa)	10^7^	10^8^
Critical tangential stress *y*_4_/(Pa)	10^7^	10^8^

To determine the critical load of the bonding model for wheat grains, we used it as the response value and designed 27 sets of experiments. Table 9 illustrates the experimental design and respective results. An analysis of these results produced a second‐order regression model that accounted for the impact of the four parameters of the wheat grain bonding model, as illustrated in Equation (6).

(6) $$\begin{array}{c} \alpha_{2}=97.73+12.962y_{1}+4.276y_{2}+1.301y_{3}-0.332y_{4}\\ \;+7.29{y_{1}}^{2}+2.07{y_{2}}^{2}-1.10{y_{3}}^{2}+2.20{y_{4}}^{2}-7.53y_{1}y_{2}\\ \;+3.19y_{1}y_{3}+0.28y_{1}y_{4}+0.34y_{2}y_{4}-1.45y_{3}y_{4} \end{array}$$

**TABLE 9:** Experimental design and experimental results.

Ber	*y*_1_	*y*_2_	*y*_3_	*y*_4_	Critical load
1	−1	−1	0	0	78.686
2	1	−1	0	0	120.740
3	−1	1	0	0	107.020
4	1	1	0	0	118.960
5	0	0	−1	−1	96.530
6	0	0	1	−1	100.860
7	0	0	−1	1	98.240
8	0	0	1	1	96.760
9	−1	0	0	−1	93.274
10	1	0	0	−1	121.020
11	−1	0	0	1	93.237
12	1	0	0	1	122.090
13	0	−1	−1	0	94.124
14	0	1	−1	0	103.660
15	0	−1	1	0	94.130
16	0	1	1	0	103.660
17	−1	0	−1	0	93.237
18	1	0	−1	0	109.330
19	−1	0	1	0	93.237
20	1	0	1	0	122.090
21	0	−1	0	−1	102.130
22	0	1	0	−1	104.290
23	0	−1	0	1	100.130
24	0	1	0	1	103.660
25	0	0	0	0	96.210
26	0	0	0	0	99.750
27	0	0	0	0	97.230

Table 10 presents the results of a variance analysis of the regression equation. The coefficient of determination, R2, of the regression equation was close to one, indicating a high degree of fit for the regression equation. Specifically, a value of *p* > .05 for the fitted model suggested that the regression equation was well fitted. The *p* values for the normal stiffness, tangential stiffness, quadratic term of the normal stiffness, and interaction term between the normal stiffness and tangential stiffness were <.0001, indicating their significant impacts on the critical load of the wheat grain bonding model. However, the critical normal stress and critical tangential stress, which had *p* values >.05, appeared to have no substantial influence on the critical load.

**TABLE 10:** ANOVA of quadratic polynomial model of Box–Behnken test.

Source of variation	Degrees of freedom	Sum of squares	Mean square	*p*‐value probability > *F*
Model	14	2895.25	206.80	<0.0001**
*y*_1_	1	2016.03	2016.03	<0.0001**
*y*_2_	1	219.39	219.39	<0.0001**
*y*_3_	1	20.32	20.32	0.1670
*y*_4_	1	1.32	1.32	0.7140
*y*_1_ *y*_1_	1	283.27	283.27	<0.0001**
*y*_2_ *y*_2_	1	22.89	22.89	0.1450
*y*_3_ *y*_3_	1	6.40	6.40	0.4260
*y*_4_ *y*_4_	1	25.82	25.82	0.1230
*y*_1_ *y*_2_	1	226.71	226.71	<0.0001**
*y*_1_ *y*_3_	1	40.70	40.70	0.0600
*y*_1_ *y*_4_	1	0.31	0.31	0.8600
*y*_2_ *y*_3_	1	0.00	0.00	0.9990
*y*_2_ *y*_4_	1	0.47	0.47	0.8270
*y*_3_ *y*_4_	1	8.44	8.44	0.3620
Residual	12	112.78	9.40	
Lack of fit	10	106.14	10.61	0.2620
Pure error	2	6.64	3.32	
The sum	26	3008.02		

**FIGURE 10:**
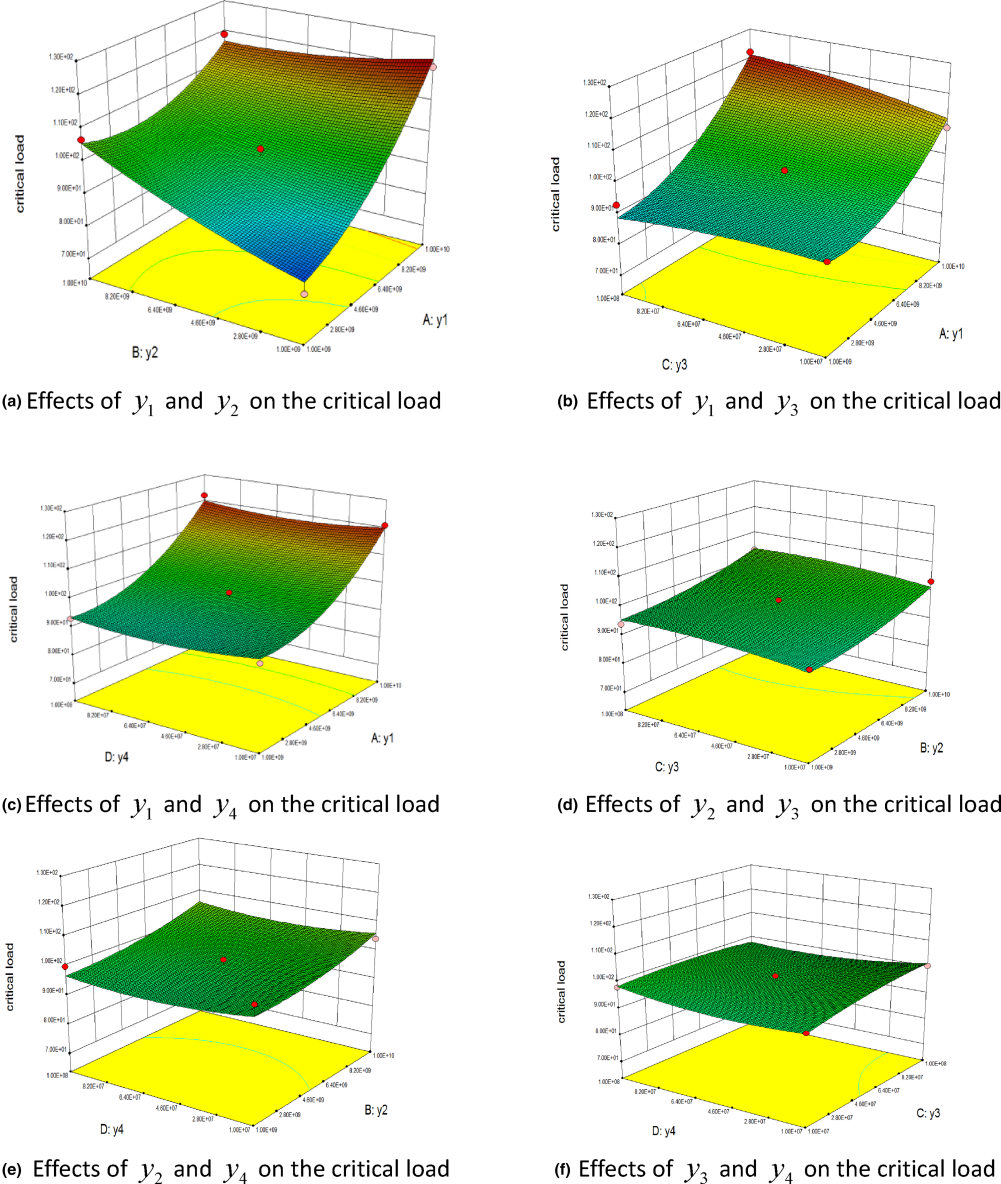
Effects of various factors on the critical load. (a) Effects of *y*_1_ and *y*_2_ on the critical load. (b) Effects of *y*_1_ and *y*_3_ on the critical load. (c) Effects of *y*_1_ and *y*_4_ on the critical load. (d) Effects of *y*_2_ and *y*_3_ on the critical load. (e) Effects of *y*_2_ and *y*_4_ on the critical load. (f) Effects of *y*_3_ and *y*_4_ on the critical load.

The results of the response surface analysis illustrated the effects of the normal contact stiffness, tangential contact stiffness, normal critical stress, and tangential critical stress on the crushing force of wheat, and the results are shown in Figure 10.

### 3.3 Model verification

With the actual critical load as the target for the response surface, the regression equation was used to calculate the optimal parameters. The optimal normal stiffness was 3.61 × 10^9^ N/m^3^; optimal tangential stiffness was 1 × 10^7^ N/m^3^; optimal critical normal stress was 1 × 10^7^ Pa; and optimal critical tangential stress was 1 × 10^8^ Pa. We input these optimal parameters into the EDEM software to validate the simulation test. After conducting five tests, we found that the error rate between the simulated critical load and regression equation was 1.6%. This low error rate confirmed the reliability of the parameter calibration and indicated that these parameters could be used for simulating the wheat‐milling process.

## 4 CONCLUSIONS

Considering the challenges observed during the wheat‐milling process, this study thoroughly investigated the recovery coefficient, as well as the static‐ and rolling‐friction coefficients for contacts between wheat particles and between the wheat and grinding roller. Additionally, we explored the influence of various factors on the wheat's angle of repose. A failure model was established to simulate the compression‐induced fracturing of wheat particles, and we also examined the impact of bond parameters on the destructive force exerted on wheat. The key findings from this research are as follows.

1. Significant contact parameters influencing the angle of repose of wheat were determined. The optimal static‐friction coefficient for the wheat–wheat interaction, rolling‐friction coefficient for the wheat–wheat interaction, and static‐friction coefficient for the wheat–grinding roller interaction were determined to be 0.3, 0.04, and 0.554, respectively. These optimal parameters were then applied in EDEM for simulation tests. After conducting five experiments, it was verified that the discrepancy between the discrete element simulation angle of repose and the actual test angle of repose was only 0.9%. This validated the reliability of the parameter calibration, which could be used to examine the wheat‐milling process and determine the simulation parameters.
2. The quadratic regression equation of the compressive destructive force of wheat was obtained when the moisture content was 15.86%. Hence, it was shown that the normal contact stiffness and tangential stiffness play decisive roles in the compressive destructive force of wheat.
3. By taking the critical load of the wheat grain's bonding model as the response value, we obtained the regression equation of the critical load via both single‐factor and Box–Behnken experiments. The regression equation was used to calculate the following optimal parameters: a normal stiffness of 3.61 × 10^9^ N/m^3^, tangential stiffness of 1 × 10^9^ N/m^3^, critical normal stress of 1 × 10^7^ Pa, and critical tangential stress of 1 × 10^8^ Pa. The optimal parameters were input into the EDEM model for calculation. The results demonstrated that the relative error between the simulated results and measured values was 1.6%. This indicated that the calibration method was both correct and feasible, and that the calibrated parameters were accurate.

## AUTHOR CONTRIBUTIONS

**Xuefeng Wang:** Resources (lead); software (lead); supervision (lead); validation (lead); visualization (lead); writing – original draft (lead); writing – review and editing (lead). **Wenbing Wu:** Conceptualization (lead); data curation (lead); formal analysis (lead); funding acquisition (lead); supervision (equal). **Huapo Jia:** Investigation (lead); methodology (lead); project administration (lead).

## CONFLICT OF INTEREST STATEMENT

The authors declare that they have no known competing financial interests or personal relationships that could have appeared to influence the work reported in this paper.

## Data Availability

All relevant data are provided in the paper.
